# Highly sensitive atomic based MW interferometry

**DOI:** 10.1038/s41598-018-27011-1

**Published:** 2018-06-06

**Authors:** Dangka Shylla, Elijah Ogaro Nyakang’o, Kanhaiya Pandey

**Affiliations:** Department of Physics, Indian Institute of Technology Guwahati, Guwahati, Assam 781039 India

## Abstract

We theoretically study a scheme to develop an atomic based micro-wave (MW) interferometry using the Rydberg states in Rb. Unlike the traditional MW interferometry, this scheme is not based upon the electrical circuits, hence the sensitivity of the phase and the amplitude/strength of the MW field is not limited by the Nyquist thermal noise. Further, this system has great advantage due to its much higher frequency range in comparision to the electrical circuit, ranging from radio frequency (RF), MW to terahertz regime. In addition, this is two orders of magnitude more sensitive to field strength as compared to the prior demonstrations on the MW electrometry using the Rydberg atomic states. Further, previously studied atomic systems are only sensitive to the field strength but not to the phase and hence this scheme provides a great opportunity to characterize the MW completely including the propagation direction and the wavefront. The atomic based MW interferometry is based upon a six-level loopy ladder system involving the Rydberg states in which two sub-systems interfere constructively or destructively depending upon the phase between the MW electric fields closing the loop. This work opens up a new field i.e. atomic based MW interferometry replacing the conventional electrical circuit in much superior fashion.

## Introduction

Atomic based standards such as time and length is already adopted and established due to their high reproducibility, accuracy, resolution and stability^1^. Atoms have also been successfully used for DC and AC (MW and RF) magnetometry, reaching impressive sensitivity and spatial resolutions^2–5^. Inspired by these successes recently, the atom based MW and RF electrometry has been investigated using the Rydberg states of the atoms^6–11^. The success of these experiments for high sensitive electrometry is due to property of the Rydberg states i.e. availability of closely spaced levels (in the range of MW and RF region) with very high electric polarizability. The strength sensitivity for MW field using the traditional antenna method is only upto 10 mV/cm^12,13^ which is limited by the thermal noise. The sensitivity is improved upto 30 *μ*V/cm using the optical method for the electro-magnetic fields converted by the dipole antenna^8,14^. The atomic based MW sensor improves the sensitivity further upto 8 *μ*V/cm^8^ which is limited by the natural decay rate of the ground and the Rydberg states, lasers linewidth, the transit time broadening, and Doppler mismatch between probe and the control lasers. The transit time broadening can be removed completely using the cold atomic cloud, cold atomic beam^15^, or nano cell^16^. The Doppler mismatch between probe and the control laser can be removed using the cold atom, nano cell or collimated atomic beam. However, with very simple experimental set-up with Rb cell at room temperature, the strength sensitivity of experimentally demonstrated four level system^8^ is already three orders of magnitude better than the electrical circuit based MW sensor. Further the frequency range of the atomic based MW sensor is from radio frequency (RF), MW to terahertz regime. Next, the spatial resolution of the atomic based MW sensor is sub-wavelength (*λ*_*MW*_/650)^17^ which is difficult to achieve with traditional antenna method as the dimension of the antenna itself happens to be *λ*_*MW*_/2.

The atomic based electrometry is based upon the phenomenon of electromagnetically induced transparency (EIT) in which the absorption property of a probe laser is altered in the presence of control lasers and MW (or RF) field in a four level system. EIT is sensitive to the field’s strength, frequency and the polarization and so the electrometry.

An oscillating electro-magnetic field i.e. MW electric field is characterized by it’s strength/amplitude, frequency, polarization and the phase. The previously studied atomic based MW electrometry is not phase sensitive as EIT in a simple multilevel system, happens to be insensitive to the absolute phase of probe and the control fields but only it’s robustness depends upon the phase stability^18^.

Phase of the MW fields is detected using traditional MW interferometry which is based upon the electrical circuit, whose performance is greatly limited by its bandwidth and the Nyquist thermal noise^19–21^. Here, we explore a six-level loopy ladder system which replaces the traditional electrical circuits based MW interferometry by the atomic MW interferometry, as the absorption property of the probe laser has phase dependency on the MW fields. This is based upon the interference between two sub-systems driven by the MW fields forming the loop. The limitation of the atomic based MW interferometry is again same as in case of the atomic based MW sensor studied with four-level system^6,8^ and is not limited by the thermal noise. But this system is two orders of magnitude more sensitive to field strength (upto 80 nV/cm) in comparison to the previously explored system^6,8^ due to its loopy nature. There are loopy system which has been studied previously and has phase sensitivity but loop is completed using the weak magnetic dipole transition^22^. In contrast to the previous system this six-level loopy system involves allowed electric dipole transition.

This paper is organized as follows. In the section namely “Method”, we describe the method of realizing the six-level loopy ladder system in Rb and possible experimental set-up. In subsequent sub-section we present the semi-classical model and solution for the relevant density matrix element. Further we provide the physical interpretation of the obtained mathematical solution in terms of the interference between the two sub-systems and in terms of the dressed state picture. In the next section namely “Results” we present various results including the lineshape of the probe absorption, the phase dependency of it, the comparison of the amplitude/strength sensitivity of this system with the previously studied four-level system and the frequency range. Finally in the section namely “Discussion” we give our conclusion for this study.

## Method

### Realization of the system

**Figure 1:**
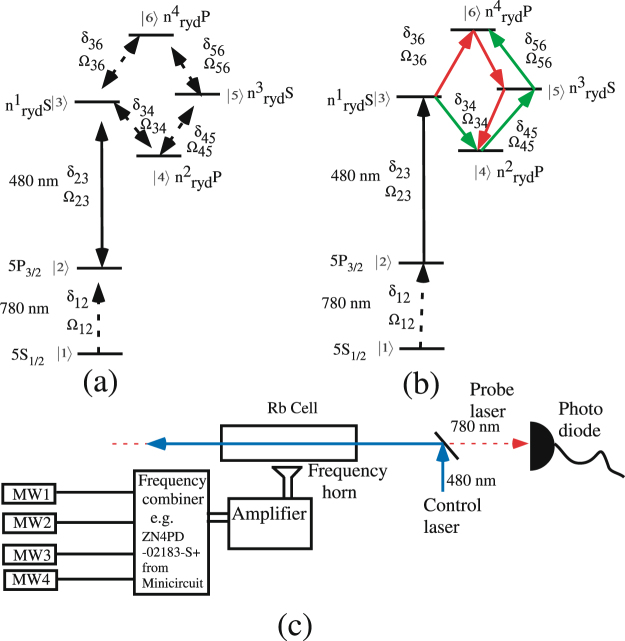
(**a**) The energy level diagram for loopy ladder system. (**b**) Transitions shown by the red and green arrow lines are the two sub-system to close the loop. The probe laser (dotted red arrow line) and the control laser (solid blue arrow line) are part of both the sub-system. (**c**) The typical experimental set up for the phase dependent MW electrometry.

The considered six-level loopy ladder system is shown in Fig. 1a. The probe laser at 780 nm is at the D_2_ line i.e. driving the 5 S_1/2_ → 5 P_3/2_ transition in the Rb. The control laser at 480 nm is driving the $5{{\rm{P}}}_{3/2}\to {{\rm{n}}}_{{\rm{ryd}}}^{1}{\rm{S}}$ and the three reference MW fields are driving the transition, ${{\rm{n}}}_{{\rm{ryd}}}^{1}{\rm{S}}\to {{\rm{n}}}_{{\rm{ryd}}}^{2}{\rm{P}}$, ${{\rm{n}}}_{{\rm{ryd}}}^{2}{\rm{P}}\to {{\rm{n}}}_{{\rm{ryd}}}^{3}{\rm{S}}$ and ${{\rm{n}}}_{{\rm{ryd}}}^{3}{\rm{S}}\to {{\rm{n}}}_{{\rm{ryd}}}^{4}{\rm{P}}$. The unknown MW field is driving the ${{\rm{n}}}_{{\rm{ryd}}}^{1}{\rm{S}}\to {{\rm{n}}}_{{\rm{ryd}}}^{4}{\rm{P}}$. The ${{\rm{n}}}_{{\rm{ryd}}}^{1}$, ${{\rm{n}}}_{{\rm{ryd}}}^{2}$, ${{\rm{n}}}_{{\rm{ryd}}}^{3}$ and ${{\rm{n}}}_{{\rm{ryd}}}^{4}$ are rydberg states which are chosen according to the frequency range of the MW field.

The typical experimental setup for phase dependent MW electrometry is shown in Fig. 1(c) in which a probe laser at 780 nm and a control laser at 480 nm are counter-propagating inside the Rb cell. The four MW control fields are generated by a single frequency synthesizer having arrangements of controlling the frequency, phase and the amplitude or the four different MW field frequencies combined using a frequency combiner (e.g. ZN4PD-02183-S+ from minicircuit company can be operated between 2–18 GHz). The output of the frequency synthesizer or combiner is amplified and fed to MW horn. All four MW fields are propagating perpendicular to the probe and the control lasers with a uniform phase inside the Rb cell.

### Semi-classical analysis

The electric field, associated with the transition |*i*〉 → |*j*〉 is ${E}_{ij}{e}^{i({\omega }_{ij}t+{\varphi }_{ij})}$, where *E*_*ij*_ is amplitude, *ω*_*ij*_ is the frequency and *ϕ*_*ij*_ is the phase. We define Rabi frequency ${{\rm{\Omega }}}_{ij}={d}_{ij}{E}_{ij}{e}^{i{\varphi }_{ij}}/\hslash$ for the transition |*i*〉 → |*j*〉 having the dipole moment matrix element *d*_*ij*_. Please note that Ω_*ij*_ is a complex quantity which can be written as |Ω_*ij*_| ${e}^{i{\varphi }_{ij}}$, where *ϕ*_*ij*_ is due to the phase of the electric field associated with it. The Rabi frequencies of the probe and the control lasers are Ω_12_ and Ω_23_ respectively, whereas ${{\rm{\Omega }}}_{34}^{{\rm{ref}}}$, ${{\rm{\Omega }}}_{45}^{{\rm{ref}}}$, ${{\rm{\Omega }}}_{56}^{{\rm{ref}}}$ and ${{\rm{\Omega }}}_{36}^{{\rm{unk}}}$ are the Rabi frequencies of the MW fields. It is important to note here that the phase of ${{\rm{\Omega }}}_{36}^{{\rm{unk}}}$ is to be characterized w.r.t to the reference MW fields ${{\rm{\Omega }}}_{34}^{{\rm{ref}}}$, ${{\rm{\Omega }}}_{45}^{{\rm{ref}}}$ and ${{\rm{\Omega }}}_{56}^{{\rm{ref}}}$. The superscript ref or unk denotes the reference and unknown MW field respectively.

The total Hamiltonian for this system is given as

1 $$\begin{array}{rcl}H & = & [\sum _{i=1}^{2}\,\frac{\hslash {{\rm{\Omega }}}_{i,i+1}}{2}({e}^{i{\omega }_{i,i+1}t}+{e}^{-i{\omega }_{i,i+1}t})|i\rangle \langle i+1|\\  &  & +\,\sum _{i=3}^{5}\,\frac{\hslash {{\rm{\Omega }}}_{i,i+1}^{{\rm{ref}}}}{2}({e}^{i{\omega }_{i,i+1}t}+{e}^{-i{\omega }_{i,i+1}t})|i\rangle \langle i+1|\\  &  & +\,\frac{\hslash {{\rm{\Omega }}}_{36}^{{\rm{unk}}}}{2}({e}^{i{\omega }_{36}t}+{e}^{-i{\omega }_{36}t})|3\rangle \langle 6|+h.c.]+\sum _{j=1}^{6}\,\hslash {\omega }_{j}|j\rangle \langle j|\end{array}$$

If the energy of the state |*i*〉 is *ℏω*_*i*_ then the general quantum mechanical state of the system is

2 $$|{\rm{\Psi }}\rangle =\sum _{i-1\,}^{6}{c}_{i}(t)|i\rangle {e}^{-i{\omega }_{i}t}$$

We define *δ*_12_ = *ω*_12_ − (*ω*_2_ − *ω*_1_) and *δ*_23_ = *ω*_23_ − (*ω*_3_ − *ω*_2_) i.e. the detunings of the probe and control lasers from their respective resonance. Similarly *δ*_34_ = *ω*_34_ − (*ω*_3_ − *ω*_4_), *δ*_45_ = *ω*_45_ − (*ω*_5_ − *ω*_4_), *δ*_56_ = *ω*_56_ − (*ω*_6_ − *ω*_5_) and *δ*_36_ = *ω*_36_ − (*ω*_6_ − *ω*_3_) are the detunings for the MW fields for the respective transitions. In the rotating frame (i.e with a unitary transformation ${c^{\prime} }_{1}={c}_{1}$; ${c^{\prime} }_{2}={c}_{2}{e}^{i{\delta }_{12}t}$; ${c^{\prime} }_{3}={c}_{3}{e}^{i({\delta }_{12}+{\delta }_{23})t}$; ${c^{\prime} }_{4}={c}_{4}{e}^{i({\delta }_{12}+{\delta }_{23}-{\delta }_{34})t}$; ${c^{\prime} }_{5}={c}_{5}{e}^{i({\delta }_{12}+{\delta }_{23}-{\delta }_{34}+{\delta }_{45})t}$; ${c^{\prime} }_{6}={c}_{6}{e}^{i({\delta }_{12}+{\delta }_{23}-{\delta }_{34}+{\delta }_{45}+{\delta }_{56})t}$) and using the rotating wave approximation, (where the terms with ${e}^{i[{\omega }_{ij}+({\omega }_{j}-{\omega }_{i})]}$ is dropped out for the transition |*i*〉 → |*j*〉 if *ω*_*j*_ > *ω*_*i*_) we get following Hamiltonian

3 $$\begin{array}{rcl}H & = & \hslash [\mathrm{0|1}\rangle \langle \mathrm{1|}-{\delta }_{12}\mathrm{|2}\rangle \langle \mathrm{2|}-({\delta }_{12}+{\delta }_{23}\mathrm{)|3}\rangle \langle \mathrm{3|}\\  &  & -\,({\delta }_{12}+{\delta }_{23}-{\delta }_{34}\mathrm{)|4}\rangle \langle \mathrm{4|}-({\delta }_{12}+{\delta }_{23}-{\delta }_{34}+{\delta }_{45}\mathrm{)|5}\rangle \langle \mathrm{5|}\\  &  & -\,({\delta }_{12}+{\delta }_{23}-{\delta }_{34}+{\delta }_{45}+{\delta }_{56})\mathrm{|6}\rangle \langle \mathrm{6|}+\frac{{{\rm{\Omega }}}_{12}}{2}\mathrm{|1}\rangle \langle \mathrm{2|}+\frac{{{\rm{\Omega }}}_{23}}{2}\mathrm{|2}\rangle \langle \mathrm{3|}\\  &  & +\,\frac{{{\rm{\Omega }}}_{34}^{{\rm{ref}}}}{2}\mathrm{|3}\rangle \langle \mathrm{4|}+\frac{{{\rm{\Omega }}}_{45}^{{\rm{ref}}}}{2}\mathrm{|4}\rangle \langle \mathrm{5|}+\frac{{{\rm{\Omega }}}_{56}^{{\rm{ref}}}}{2}\mathrm{|5}\rangle \langle \mathrm{6|}\\  &  & +\,\frac{{{\rm{\Omega }}}_{36}^{{\rm{unk}}}}{2}{e}^{i({\delta }_{34}-{\delta }_{45}-{\delta }_{56}+{\delta }_{36})t}\mathrm{|3}\rangle \langle \mathrm{6|}+h.c.]\end{array}$$

In general, the Hamilitonian *H* is time dependent except for a particular condition when *δ*_34_ − *δ*_45_ − *δ*_56_ + *δ*_36_ = 0.

The time evolution of the density matrix, *ρ* is given by Linblad master equation as

4 $$\dot{\rho }=-\,\frac{i}{\hslash }[H,\rho ]+L[\rho (t)]$$

where, *L*[*ρ*(*t*)] is Linblad matrix and defined as below. *L*[*ρ*(*t*)] =

5 $$[\begin{array}{llllll}{{\rm{\Gamma }}}_{21}{\rho }_{22} & -\tfrac{{\gamma }_{12}^{dec}}{2}{\rho }_{12} & -\tfrac{{\gamma }_{13}^{dec}}{2}{\rho }_{13} & -\tfrac{{\gamma }_{14}^{dec}}{2}{\rho }_{14} & -\tfrac{{\gamma }_{15}^{dec}}{2}{\rho }_{15} & -\tfrac{{\gamma }_{16}^{dec}}{2}{\rho }_{16}\\ -\tfrac{{\gamma }_{12}^{dec}}{2}{\rho }_{21} & -{{\rm{\Gamma }}}_{21}{\rho }_{22}+{{\rm{\Gamma }}}_{32}{\rho }_{33} & -\tfrac{{\gamma }_{23}^{dec}}{2}{\rho }_{23} & -\tfrac{{\gamma }_{24}^{dec}}{2}{\rho }_{24} & -\tfrac{{\gamma }_{25}^{dec}}{2}{\rho }_{25} & -\tfrac{{\gamma }_{26}^{dec}}{2}{\rho }_{26}\\ -\tfrac{{\gamma }_{13}^{dec}}{2}{\rho }_{31} & -\tfrac{{\gamma }_{23}^{dec}}{2}{\rho }_{32} & -{{\rm{\Gamma }}}_{32}{\rho }_{33}-{{\rm{\Gamma }}}_{34}{\rho }_{33}+{{\rm{\Gamma }}}_{63}{\rho }_{66} & -\tfrac{{\gamma }_{34}^{dec}}{2}{\rho }_{34} & -\tfrac{{\gamma }_{35}^{dec}}{2}{\rho }_{35} & -\tfrac{{\gamma }_{36}^{dec}}{2}{\rho }_{36}\\ -\tfrac{{\gamma }_{14}^{dec}}{2}{\rho }_{41} & -\tfrac{{\gamma }_{24}^{dec}}{2}{\rho }_{42} & -\tfrac{{\gamma }_{34}^{dec}}{2}{\rho }_{43} & {{\rm{\Gamma }}}_{34}{\rho }_{33}-{{\rm{\Gamma }}}_{4}{\rho }_{44} & -\tfrac{{\gamma }_{45}^{dec}}{2}{\rho }_{45} & -\tfrac{{\gamma }_{46}^{dec}}{2}{\rho }_{46}\\ -\tfrac{{\gamma }_{15}^{dec}}{2}{\rho }_{51} & -\tfrac{{\gamma }_{25}^{dec}}{2}{\rho }_{52} & -\tfrac{{\gamma }_{35}^{dec}}{2}{\rho }_{53} & -\tfrac{{\gamma }_{45}^{dec}}{2}{\rho }_{54} & -{{\rm{\Gamma }}}_{5}{\rho }_{55} & -\tfrac{{\gamma }_{56}^{dec}}{2}{\rho }_{56}\\ -\tfrac{{\gamma }_{16}^{dec}}{2}{\rho }_{61} & -\tfrac{{\gamma }_{26}^{dec}}{2}{\rho }_{62} & -\tfrac{{\gamma }_{36}^{dec}}{2}{\rho }_{63} & -\tfrac{{\gamma }_{46}^{dec}}{2}{\rho }_{64} & -\tfrac{{\gamma }_{56}^{dec}}{2}{\rho }_{65} & -{{\rm{\Gamma }}}_{6}{\rho }_{66}\end{array}]$$

Where, Γ_*ij*_ is the decay of the population from state |*i*〉 (*i* = 1, 2, .. to 6) to state |*j*〉 (*j* = 1, 2, .. 6) and Γ_*i*_ is the total population decay rate of state |*i*〉. In the case of the weak probe, the population transfer does not take place and it is completely irrelevant to know the population dynamics between different levels. The only important parameter is Γ_*i*_ and Γ_*j*_, i.e. the total decay rate of states, which governs the decoherence rate (${\gamma }_{ij}^{dec}$) between the two levels |*i*〉 and |*j*〉 as ${\gamma }_{ij}^{dec}=\frac{{{\rm{\Gamma }}}_{i}+{{\rm{\Gamma }}}_{j}}{2}$. In addition to the total decay rate of states, the linewidth of lasers driving the transition has to be also included for ${\gamma }_{ij}^{dec}$. For example, in this study we take the value of ${\gamma }_{12}^{dec}=2\pi \times 3.05\,{\rm{MHz}}$, which includes natural radiative decay of excited state, Γ_2_ = 2*π* × 6 MHz and the 780 nm laser linewidth of 2*π* × 50 kHz. We also take ${\gamma }_{13}^{dec}={\gamma }_{14}^{dec}={\gamma }_{15}^{dec}={\gamma }_{16}^{dec}={\gamma }^{dec}=2\pi \times 100\,{\rm{kHz}}$ mainly dominated by the laser linewidths of 780 nm and the 480 nm as compared to the radiative decay rate (=2*π* × 1 kHz) of the Rydberg states |3〉, |4〉, |5〉 and |6〉^7^. We also take *γ*^*dec*^ = 2*π* × 500 kHz in some cases in order to check it’s stringency.

From Eqs 3, 4 and 5 we get 36 coupled differential equations with the property ${\rho }_{ij}={\rho }_{ji}^{\ast }$. In order to solve these set of coupled equation we adapt similar method as in the case of previously studied multi-level systems^23^.

In the case of weak probe approximation, there will be no population transfer and hence the time evolution of the population i.e. the diagonal terms of the density matrix such as *ρ*_11_, *ρ*_22_, *ρ*_33_, *ρ*_44_, *ρ*_55_, and *ρ*_66_ can be ignored. Similarly the time evolution of the off-diagonal terms *ρ*_*ij*_ for *i* = 2; *j* = 3, 4, 5, 6 and *i* = 3; *j* = 4, 5, 6 and *i* = 4; *j* = 5, 6 and *i* = 5; *j* = 6 can be also ignored. The time evolution of the relevant density matrix element is given below.

6 $$\begin{array}{rcl}{\dot{\rho }}_{12} & = & i\frac{{{\rm{\Omega }}}_{12}}{2}({\rho }_{11}-{\rho }_{22})+i\frac{{{\rm{\Omega }}}_{23}^{\ast }}{2}{\rho }_{13}-{\gamma }_{12}{\rho }_{12}\\ {\dot{\rho }}_{13} & = & -i\frac{{{\rm{\Omega }}}_{12}}{2}{\rho }_{23}+i\frac{{{\rm{\Omega }}}_{23}}{2}{\rho }_{12}+i\frac{{{\rm{\Omega }}}_{34}^{{{\rm{ref}}}^{\ast }}}{2}{\rho }_{14}\\  &  & +\,i\frac{{{\rm{\Omega }}}_{36}^{{{\rm{unk}}}^{\ast }}}{2}{e}^{-i({\delta }_{34}-{\delta }_{45}-{\delta }_{56}+{\delta }_{36})t}{\rho }_{16}-{\gamma }_{13}{\rho }_{13}\\ {\dot{\rho }}_{14} & = & -i\frac{{{\rm{\Omega }}}_{12}}{2}{\rho }_{24}+i\frac{{{\rm{\Omega }}}_{34}^{{\rm{ref}}}}{2}{\rho }_{13}+i\frac{{{\rm{\Omega }}}_{45}^{{{\rm{ref}}}^{\ast }}}{2}{\rho }_{15}-{\gamma }_{14}{\rho }_{14}\\ {\dot{\rho }}_{15} & = & -i\frac{{{\rm{\Omega }}}_{12}}{2}{\rho }_{25}+i\frac{{{\rm{\Omega }}}_{45}^{{\rm{ref}}}}{2}{\rho }_{14}+i\frac{{{\rm{\Omega }}}_{56}^{{{\rm{ref}}}^{\ast }}}{2}{\rho }_{16}-{\gamma }_{15}{\rho }_{15}\\ {\dot{\rho }}_{16} & = & -i\frac{{{\rm{\Omega }}}_{12}}{2}{\rho }_{26}+i\frac{{{\rm{\Omega }}}_{36}^{{\rm{unk}}}}{2}{e}^{i({\delta }_{34}-{\delta }_{45}-{\delta }_{56}+{\delta }_{36})t}{\rho }_{13}+i\frac{{{\rm{\Omega }}}_{56}^{{\rm{ref}}}}{2}{\rho }_{15}-{\gamma }_{16}{\rho }_{16}\end{array}$$

Where, $${\gamma }_{12}=[{\gamma }_{12}^{dec}+i{\delta }_{12}]$$,

$$\begin{array}{rcl}{\gamma }_{13} & = & [{\gamma }_{13}^{dec}+i({\delta }_{12}+{\delta }_{23})],\\ {\gamma }_{14} & = & [{\gamma }_{14}^{dec}+i({\delta }_{12}+{\delta }_{23}-{\delta }_{34})],\\ {\gamma }_{15} & = & [{\gamma }_{15}^{dec}+i({\delta }_{12}+{\delta }_{23}-{\delta }_{34}+{\delta }_{45})],\\ {\gamma }_{16} & = & [{\gamma }_{16}^{dec}+i({\delta }_{12}+{\delta }_{23}-{\delta }_{34}+{\delta }_{45}+{\delta }_{56})],\end{array}$$

**Figure 2:**
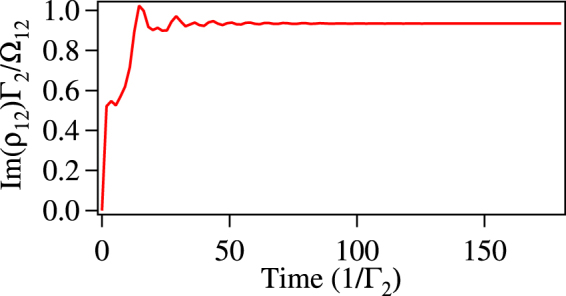
The normalized absorption, *ρ*_12_Γ_2_/Ω_12_ vs Time for ${{\rm{\Omega }}}_{23}={{\rm{\Omega }}}_{34}^{{\rm{ref}}}={{\rm{\Omega }}}_{45}^{{\rm{ref}}}={{\rm{\Omega }}}_{56}^{{\rm{ref}}}={{\rm{\Gamma }}}_{2}$, ${{\rm{\Omega }}}_{36}^{{\rm{ukn}}}=0.5{{\rm{\Gamma }}}_{2}$ and *δ*_12_ = *δ*_23_ = *δ*_34_ = *δ*_45_ = *δ*_56_ = *δ*_36_ = 0.

Now, we apply the four-photon resonance condition for the MW fields i.e. *δ*_34_ − *δ*_45_ − *δ*_56_ + *δ*_36_ = 0. In this case the system will reach steady state i.e. ${\dot{\rho }}_{ij}=0$, for all the elements on the time scale of few tens of 1/Γ_2_ as shown in Fig. 2. In the weak probe condition and in the steady state, *ρ*_11_ ≈ 1, *ρ*_22_ ≈ *ρ*_33_ ≈ *ρ*_44_ ≈ *ρ*_55_ ≈ *ρ*_66_ ≈ 0 and *ρ*_*ij*_ = *ρ*_*ji*_ ≈ 0 for *i* = 2; *j* = 3, 4, 5, 6 and *i* = 3; *j* = 4, 5, 6 and *i* = 4; *j* = 5, 6 and *i* = 5; *j* = 6. Finally, we get the following set of equations

7 $$\begin{array}{rcl}{\rho }_{12} & = & \frac{i}{2}\frac{{{\rm{\Omega }}}_{12}}{{\gamma }_{12}}+\frac{i}{2}\frac{{{\rm{\Omega }}}_{23}^{\ast }}{{\gamma }_{12}}{\rho }_{13}\\ {\rho }_{13} & = & \frac{i}{2}\frac{{{\rm{\Omega }}}_{23}}{{\gamma }_{13}}{\rho }_{12}+\frac{i}{2}\frac{{{\rm{\Omega }}}_{34}^{{{\rm{ref}}}^{\ast }}}{{\gamma }_{13}}{\rho }_{14}+\frac{i}{2}\frac{{{\rm{\Omega }}}_{36}^{{{\rm{unk}}}^{\ast }}}{{\gamma }_{13}}{\rho }_{16}\\ {\rho }_{14} & = & \frac{i}{2}\frac{{{\rm{\Omega }}}_{34}^{{\rm{ref}}}}{{\gamma }_{14}}{\rho }_{13}+\frac{i}{2}\frac{{{\rm{\Omega }}}_{45}^{{{\rm{ref}}}^{\ast }}}{{\gamma }_{14}}{\rho }_{15}\\ {\rho }_{15} & = & \frac{i}{2}\frac{{{\rm{\Omega }}}_{45}^{{\rm{ref}}}}{{\gamma }_{15}}{\rho }_{14}+\frac{i}{2}\frac{{{\rm{\Omega }}}_{56}^{{{\rm{ref}}}^{\ast }}}{{\gamma }_{15}}{\rho }_{16}\\ {\rho }_{16} & = & \frac{i}{2}\frac{{{\rm{\Omega }}}_{36}^{{\rm{unk}}}}{{\gamma }_{16}}{\rho }_{13}+\frac{i}{2}\frac{{{\rm{\Omega }}}_{56}^{{\rm{ref}}}}{{\gamma }_{16}}{\rho }_{15}\end{array}$$

The above equation gives solution for *ρ*_12_ as

$${\rho }_{12}=\tfrac{\tfrac{i}{2}\tfrac{{{\rm{\Omega }}}_{12}}{{\gamma }_{12}}}{1+\tfrac{\tfrac{1}{4}\tfrac{|{{\rm{\Omega }}}_{23}{|}^{2}}{{\gamma }_{12}{\gamma }_{13}}}{1+{\rm{EITATA}}1+{\rm{EITATA}}2+\,{\rm{Int}}}}$$

where,

8 $$\begin{array}{rcl}{\rm{EITATA}}1 & = & \frac{\frac{1}{4}\frac{|{{\rm{\Omega }}}_{34}^{{\rm{ref}}}{|}^{2}}{{\gamma }_{13}{\gamma }_{14}}}{1+\frac{\frac{1}{4}\frac{|{{\rm{\Omega }}}_{45}^{{\rm{ref}}}{|}^{2}}{{\gamma }_{14}{\gamma }_{15}}}{1+\frac{1}{4}\frac{|{{\rm{\Omega }}}_{56}^{{\rm{ref}}}{|}^{2}}{{\gamma }_{15}{\gamma }_{16}}}};\\ {\rm{EITATA}}2 & = & \frac{\frac{1}{4}\frac{|{{\rm{\Omega }}}_{36}^{{\rm{unk}}}{|}^{2}}{{\gamma }_{13}{\gamma }_{16}}}{1+\frac{\frac{1}{4}\frac{|{{\rm{\Omega }}}_{56}^{{\rm{ref}}}{|}^{2}}{{\gamma }_{15}{\gamma }_{16}}}{1+\frac{1}{4}\frac{|{{\rm{\Omega }}}_{45}^{{\rm{ref}}}{|}^{2}}{{\gamma }_{14}{\gamma }_{15}}}};\\ {\rm{Int}} & = & -\frac{\frac{1}{8}\frac{|{{\rm{\Omega }}}_{34}^{{\rm{ref}}}||{{\rm{\Omega }}}_{45}^{{\rm{ref}}}||{{\rm{\Omega }}}_{56}^{{\rm{ref}}}||{{\rm{\Omega }}}_{36}^{{\rm{unk}}}|cos(\varphi )}{{\gamma }_{13}{\gamma }_{14}{\gamma }_{15}{\gamma }_{16}}}{1+\frac{1}{4}\frac{|{{\rm{\Omega }}}_{45}^{{\rm{ref}}}{|}^{2}}{{\gamma }_{14}{\gamma }_{15}}+\frac{1}{4}\frac{|{{\rm{\Omega }}}_{56}^{{\rm{ref}}}{|}^{2}}{{\gamma }_{15}{\gamma }_{16}}};\\ \varphi  & = & {\varphi }_{36}^{{\rm{unk}}}-{\varphi }_{34}^{{\rm{ref}}}-{\varphi }_{45}^{{\rm{ref}}}-{\varphi }_{56}^{{\rm{ref}}}\end{array}$$

The refractive index, *n* of the probe laser is related with the density matrix element, *ρ*_12_ as $n=1+3{{\rm{\lambda }}}_{p}^{2}N\mathrm{/(2}\pi )({{\rm{\Gamma }}}_{2}/{{\rm{\Omega }}}_{12}){\rho }_{12}$, where *λ*_*p*_(=780 nm) is the wavelength of the probe laser and *N* is atomic number density^24,25^. The imaginary part of *n* is related with the absorption and real part with dispersion. We define the normalized absorption [(Γ_2_/Ω_12_) Im(*ρ*_12_)] i.e. for the stationary atoms, the absorption of the probe laser at resonance in the absence of all the control lasers is 1.

**Figure 3:**
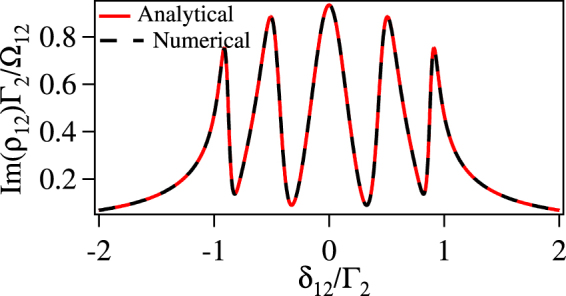
Comparison of complete numerical solution with the analytical solution for the normalized absorption (Im(*ρ*_12_)Γ_2_/Ω_12_) vs *δ*_12_/Γ_2_ of the probe laser with $|{{\rm{\Omega }}}_{23}|=|{{\rm{\Omega }}}_{34}^{{\rm{ref}}}|=|{{\rm{\Omega }}}_{45}^{{\rm{ref}}}|=|{{\rm{\Omega }}}_{56}^{{\rm{ref}}}|={{\rm{\Gamma }}}_{2}$, $|{{\rm{\Omega }}}_{36}^{{\rm{unk}}}|=0.5{{\rm{\Gamma }}}_{2}$, *ϕ* = 0 and *δ*_23_ = *δ*_34_ = *δ*_45_ = *δ*_56_ = *δ*_36_ = 0.

In order to verify the approximation made above, we have checked the analytical solution of *ρ*_12_ given by the Eq. 8 and the complete numerical solution in the steady state for various values of control fields and detunings. It has excellent agreement between complete numerical and approximated analytical solution as shown in Fig. 3. The solution for *ρ*_12_ in Eq. 8 has the following interpretation.

### Interpretation

#### Interference between two sub-system

Equation 8 looks very complicated but it can be interpreted in the following simple way. The closed loop system can be realized by two open loop sub-systems |3〉 → |4〉 → |5〉 → |6〉 and |3〉 → |6〉 → |5〉 → |4〉 shown with red and green arrows respectively as shown in Fig. 1b. These two sub-system shares a common |1〉 → |2〉 → |3〉 ladder system. In order to understand the absorption property of the probe laser Ω_12_, we switch on the control fields one by one and in the sequence for the two sub-systems. Firstly, the control laser Ω_23_ causes transparency for the probe laser Ω_12_ and known as EIT. For path shown with the red color, the control field ${{\rm{\Omega }}}_{34}^{{\rm{ref}}}$ recovers the absorption against the EIT created by Ω_23_ and known as EITA. Again the control fields ${{\rm{\Omega }}}_{45}^{{\rm{ref}}}$ causes transparency against the EITA created by the Ω_23_ and ${{\rm{\Omega }}}_{34}^{{\rm{ref}}}$, and known as EITAT. Finally the ${{\rm{\Omega }}}_{56}^{{\rm{ref}}}$ causes absorption against the EITAT created by the Ω_23_, ${{\rm{\Omega }}}_{34}^{{\rm{ref}}}$ and ${{\rm{\Omega }}}_{45}^{{\rm{ref}}}$, and known as EITATA^23^ and expressed by EITATA1 in Eq. 8. (In order to understand the transparency and absorption in the sequence, we strongly advice the readers to see the paper^23^). The other path shown with green color will also cause EITATA by sequence of the control fields ${{\rm{\Omega }}}_{36}^{{\rm{unk}}}$, ${{\rm{\Omega }}}_{56}^{{\rm{ref}}}$ and ${{\rm{\Omega }}}_{45}^{{\rm{ref}}}$ which is expressed by EITATA2. Further, these two sub-system causing EITATA1 and EITATA2, interferes with each other and expressed by the Int term in the Eq. 8, which is phase(*ϕ*) dependent.

**Figure 4:**
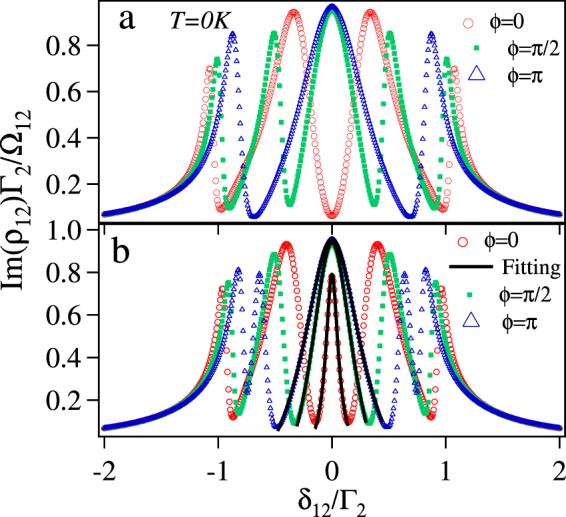
Normalized absorption (Im(*ρ*_12_)Γ_2_/Ω_12_) vs *δ*_12_/Γ_2_ of the probe laser with $$|{{\rm{\Omega }}}_{23}|=|{{\rm{\Omega }}}_{34}^{{\rm{ref}}}|=|{{\rm{\Omega }}}_{45}^{{\rm{ref}}}|=$$$$|{{\rm{\Omega }}}_{56}^{{\rm{ref}}}|={{\rm{\Gamma }}}_{2}$$, *δ*_23_ = *δ*_34_ = *δ*_45_ = *δ*_56_ = *δ*_36_ = 0 and (**a**) $$|{{\rm{\Omega }}}_{36}^{{\rm{unk}}}|={{\rm{\Gamma }}}_{2}$$ (**b**) $$|{{\rm{\Omega }}}_{36}^{{\rm{unk}}}|=0.5{{\rm{\Gamma }}}_{2}$$.

In the other words, the closed loop |3〉 → |4〉 → |5〉 → |6〉 → |3〉 causes absorption against EIT created by the control laser Ω_23_. The closed loop has two-open loop sub-systems which interfere destructively (for *ϕ* = 0) and constructively (for *ϕ* = *π*) with each other. As shown in Fig. 4a, for $|{{\rm{\Omega }}}_{34}^{{\rm{ref}}}|=|{{\rm{\Omega }}}_{45}^{{\rm{ref}}}|=|{{\rm{\Omega }}}_{56}^{{\rm{ref}}}|=|{{\rm{\Omega }}}_{36}^{{\rm{unk}}}|={{\rm{\Gamma }}}_{2}(\,\gg \,{\gamma }^{dec})$, there is a complete transparency at the line center for *ϕ* = 0. This is due to perfect destructive interference between the two-subsystems as the strength is same for both, i.e. EITATA1 = EITATA2. There is maxi-mum absorption at the line center for *ϕ* = *π* as the two sub-systems are interfering constructively. For $|{{\rm{\Omega }}}_{34}^{{\rm{ref}}}|=|{{\rm{\Omega }}}_{45}^{{\rm{ref}}}|=|{{\rm{\Omega }}}_{56}^{{\rm{ref}}}|\ne |{{\rm{\Omega }}}_{36}^{{\rm{unk}}}|\gg {\gamma }^{dec}$, there is a absorption peak at the line center for *ϕ* = 0, as shown in Fig. 4b. This is due to unequal strength of the individual system (EITATA1 > EITATA2), hence the destructive interference between them is not perfect.

#### Dressed state approach

At high Rabi frequencies (much greater than the absorption peaks linewidths) of the control lasers and MW fields, the linewidth of the absorption peak can be explained using dressed state picture. In this condition there is no interference between the absorption peaks as they are well separated from each other. The position of the absorption peak is determined by the eigenvalues of the Hamiltonian associated to the control fields as given below

9 $$\begin{array}{rcc}{H}_{c} & = & [\begin{array}{ccccc}0 & \frac{{{\rm{\Omega }}}_{23}}{2} & 0 & 0 & 0\\ \frac{{{\rm{\Omega }}}_{23}^{\ast }}{2} & {\delta }_{23} & \frac{|{{\rm{\Omega }}}_{34}^{{\rm{ref}}}|}{2}{e}^{i{\varphi }_{34}} & 0 & \frac{|{{\rm{\Omega }}}_{36}^{{\rm{unk}}}|}{2}{e}^{i{\varphi }_{36}}\\ 0 & \frac{|{{\rm{\Omega }}}_{34}^{{\rm{ref}}}|}{2}{e}^{-i{\varphi }_{34}} & {\delta }_{23}-{\delta }_{34} & \frac{|{{\rm{\Omega }}}_{45}^{{\rm{ref}}}|}{2}{e}^{i{\varphi }_{45}} & 0\\ 0 & 0 & \frac{|{{\rm{\Omega }}}_{45}^{{\rm{ref}}}|}{2}{e}^{-i{\varphi }_{45}} & {\delta }_{23}-{\delta }_{34}+{\delta }_{45} & \frac{|{{\rm{\Omega }}}_{56}^{{\rm{ref}}}|}{2}{e}^{i{\varphi }_{56}}\\ 0 & \frac{|{{\rm{\Omega }}}_{36}^{{\rm{unk}}}|}{2}{e}^{-i{\varphi }_{36}} & 0 & \frac{|{{\rm{\Omega }}}_{56}^{{\rm{ref}}}|}{2}{e}^{-i{\varphi }_{56}} & {\delta }_{23}-{\delta }_{34}+{\delta }_{45}+{\delta }_{56}\end{array}]\end{array}$$

For general control fields detunings and Rabi frequencies, the position of the absorption peaks will be complicated. However, the expression becomes simpler for zero detuning of control fields and with $$|{{\rm{\Omega }}}_{23}|=|{{\rm{\Omega }}}_{34}^{{\rm{ref}}}|=|{{\rm{\Omega }}}_{45}^{{\rm{ref}}}|=|{{\rm{\Omega }}}_{56}^{{\rm{ref}}}|={\rm{\Omega }}$$, but with arbitrary values of $$|{\Omega }_{36}^{{\rm{unk}}}|$$. In this condition the positions of the absorption peaks (i.e. eigenvalues of the *H*_*c*_) are −$$\frac{1}{\sqrt{8}}$$$$\sqrt{4{{\rm{\Omega }}}^{2}+{|{\rm{\Omega }}}_{36}^{{\rm{unk}}}{|}^{2}+\sqrt{{(2{{\rm{\Omega }}}^{2}+{|{\rm{\Omega }}}_{36}^{{\rm{unk}}}{|}^{2})}^{2}+8{{\rm{\Omega }}}^{3}{|{\rm{\Omega }}}_{36}^{{\rm{unk}}}|cos\varphi }}$$, −$$\frac{1}{\sqrt{8}}$$$$\sqrt{4{{\rm{\Omega }}}^{2}+{|{\rm{\Omega }}}_{36}^{{\rm{unk}}}{|}^{2}-\sqrt{{(2{{\rm{\Omega }}}^{2}+{|{\rm{\Omega }}}_{36}^{{\rm{unk}}}{|}^{2})}^{2}+8{{\rm{\Omega }}}^{3}{|{\rm{\Omega }}}_{36}^{{\rm{unk}}}|cos\varphi }}$$, 0, $$\frac{1}{\sqrt{8}}$$$$\sqrt{4{{\rm{\Omega }}}^{2}+{|{\rm{\Omega }}}_{36}^{{\rm{unk}}}{|}^{2}-\sqrt{{(2{{\rm{\Omega }}}^{2}+{|{\rm{\Omega }}}_{36}^{{\rm{unk}}}{|}^{2})}^{2}+8{{\rm{\Omega }}}^{3}{|{\rm{\Omega }}}_{36}^{{\rm{unk}}}|cos\varphi }}$$, and $$\frac{1}{\sqrt{8}}$$$$\sqrt{4{{\rm{\Omega }}}^{2}+{|{\rm{\Omega }}}_{36}^{{\rm{unk}}}{|}^{2}+\sqrt{{(2{{\rm{\Omega }}}^{2}+{|{\rm{\Omega }}}_{36}^{{\rm{unk}}}{|}^{2})}^{2}+8{{\rm{\Omega }}}^{3}{|{\rm{\Omega }}}_{36}^{{\rm{unk}}}|cos\varphi }}$$.

The eigenvectors determines the dressed state in terms of the bare atomic states. For example the normalized eigenvector corresponding to eigenvalue 0 is

10 $$\frac{1}{[{(1+\tfrac{|{{\rm{\Omega }}}_{36}^{{\rm{unk}}}{|}^{2}}{{{\rm{\Omega }}}^{2}}-2\tfrac{|{{\rm{\Omega }}}_{36}^{{\rm{unk}}}|}{{\rm{\Omega }}}cos\varphi )+2]}^{\mathrm{1/2}}]}[\begin{array}{c}1-\frac{|{{\rm{\Omega }}}_{36}^{{\rm{unk}}}|}{{\rm{\Omega }}}{e}^{i\varphi }\\ 0\\ -\,1\\ 0\\ 1\end{array}]$$

This is the central dressed state (or the central absorption peak) and is expressed as $$[(1-\tfrac{|{{\rm{\Omega }}}_{36}^{{\rm{unk}}}|{e}^{i\varphi }}{{\rm{\Omega }}})$$$$|2\rangle -|4\rangle +|6\rangle ]$$/$${[(1+\tfrac{{|{{\rm{\Omega }}}_{36}^{{\rm{unk}}}|}^{2}}{{{\rm{\Omega }}}^{2}}-2\tfrac{|{{\rm{\Omega }}}_{36}^{{\rm{unk}}}|}{{\rm{\Omega }}}cos\varphi )+2]}^{\mathrm{1/2}}$$. The linewidth of the dressed state or the absorption peak is given in terms of the bare atomic states decay rate. For example, if dressed state is written as *C*_2_|2〉 + *C*_3_|3〉 + *C*_4_|4〉 + *C*_5_|5〉 then the linewidth of it will be |*C*_2_|^2^Γ_2_ + |*C*_3_|^2^Γ_3_ + |*C*_4_|^2^Γ_4_ + |*C*_5_|^2^Γ_5_ Hence the linewidth of the cenetral absorption peak is given by $$[(1+\tfrac{{|{{\rm{\Omega }}}_{36}^{{\rm{unk}}}|}^{2}}{{{\rm{\Omega }}}^{2}}-2\tfrac{{{\rm{\Omega }}}_{36}^{{\rm{unk}}}}{{\rm{\Omega }}}cos\varphi ){{\rm{\Gamma }}}_{2}+{{\rm{\Gamma }}}_{4}+{{\rm{\Gamma }}}_{6}]$$/$$[(1+\tfrac{|{{\rm{\Omega }}}_{36}^{{\rm{unk}}}|}{{{\rm{\Omega }}}^{2}}-2\tfrac{{{\rm{\Omega }}}_{36}^{{\rm{unk}}}}{{\rm{\Omega }}}cos\varphi )+2]$$ which is phase dependent. In order to crosscheck the expression for the linewidth, we fit (shown with black solid line) the central peak of the normalized absorption obtained by Eq. 8 with Lorentzian profile to find the linewidth for three different phases as shown in Fig. 4. The fitted linewidths for *ϕ* = 0, *ϕ* = *π*/2 and *ϕ* = *π* are 0.13Γ_2_, 0.47Γ_2_ and 0.64Γ_2_ respectively, while the calculated linewidths are 0.13Γ_2_, 0.39Γ_2_ and 0.54Γ_2_ respectively. There is a small mismatch between the fitted and the calculated linewidths by the dressed state approach for *ϕ* = *π*/2 and *ϕ* = *π*. This is because, as we see in Fig. 4, the central absorption peak is broadened for *ϕ* = *π*/2 and *ϕ* = *π* and the interference between peaks starts playing a role in the modification of the linewidth similar to three level system^26^.

## Results

### Probe laser absorption

The normalized absorption (Im(*ρ*_12_)Γ_2_/Ω_12_) vs probe detuning (*δ*_12_) for three different phases, *ϕ* = 0,*π*/2 and *π* is shown in Fig. 4. For the central absorption peak i.e. at *δ*_12_ = 0, only the linewidth depends upon the phase but not the position, while both the position and the linewidth depends upon the phase(*ϕ*) for the other four absorption peaks. This has been explained in the previous section.

**Figure 5:**
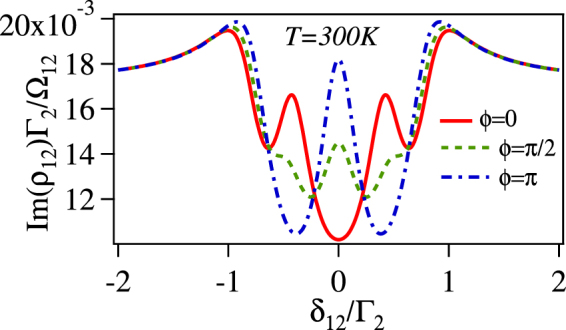
Normalized absorption of the probe laser with thermal averaging (Im(${\rho }_{12}^{{\rm{Thermal}}}$)Γ_2_/Ω_12_) vs *δ*_12_/Γ_2_ with $|{{\rm{\Omega }}}_{23}|=|{{\rm{\Omega }}}_{34}^{{\rm{ref}}}|=|{{\rm{\Omega }}}_{45}^{{\rm{ref}}}|=|{{\rm{\Omega }}}_{56}^{{\rm{ref}}}|={{\rm{\Gamma }}}_{2}$, $|{{\rm{\Omega }}}_{36}^{{\rm{unk}}}|=0.5{{\rm{\Gamma }}}_{2}$ and *δ*_23_ = *δ*_34_ = *δ*_45_ = *δ*_56_ = *δ*_36_ = 0.

Now, we consider the effect of the temperature as lineshape of EIT is significantly changed by the thermal averaging^27–32^. The thermal averaging of *ρ*_12_ is done numerically for the room temperature (*T* = 300 K) for the counter-propagating configuration of the probe (Ω_12_) and the control laser (Ω_23_) with wave-vectors *k*_780_ and *k*_480_ respectively by replacing *δ*_12_ with *δ*_12_ + *k*_780_*v* and *δ*_23_ with *δ*_23_ − *k*_480_*v* for moving atoms with velocity *v*, while the Doppler shift for the MW fields are ignored. Further the *ρ*_12_ is weighted by the Maxwell Boltzman velocity distribution function and integrated over the velocity as ${\rho }_{12}^{{\rm{Thermal}}}=\sqrt{\frac{m}{2\pi {k}_{B}T}}\int {\rho }_{12}(v){e}^{-\frac{m{v}^{2}}{2{k}_{B}T}}dv$, where *k*_*B*_ is Boltzman constant and *m* is atomic mass of Rb. The integration is done over velocity range which is three times of $\sqrt{\frac{{k}_{B}T}{m}}$. The Doppler averaging changes the absorption profile significantly as shown in Fig. 5. One of the interesting modification is the phase dependency of the probe laser absorption at the zero detunings of the probe. The probe laser absorption is minimum for *ϕ* = 0 and maximum for *ϕ* = *π* as shown with red and blue curve respectively in Fig. 5. This modification is due to mismatch of Doppler shift for probe at 780 nm and the control at 480 nm for moving atom. Please note that without thermal averaging at zero detunings of the probe, control laser and MW fields, probe laser absorption has no significant difference between *ϕ* = *π*/2 and *π*.

### Phase sensitivity

#### Sinusoidal behavior

As seen in the previous section that the absorption profile of the probe laser depends upon the phase, *ϕ*. Please note that the previously studied (i.e. four-level) system^6–11^ were insensitive to the phase of the MW field. This is also clear from Eq. 8 in the special case with $|{{\rm{\Omega }}}_{34}^{{\rm{ref}}}|=|{{\rm{\Omega }}}_{45}^{{\rm{ref}}}|=|{{\rm{\Omega }}}_{56}^{{\rm{ref}}}|=0$, which reduces the six-level loopy ladder system to four-level system and will have no phase dependency.

**Figure 6:**
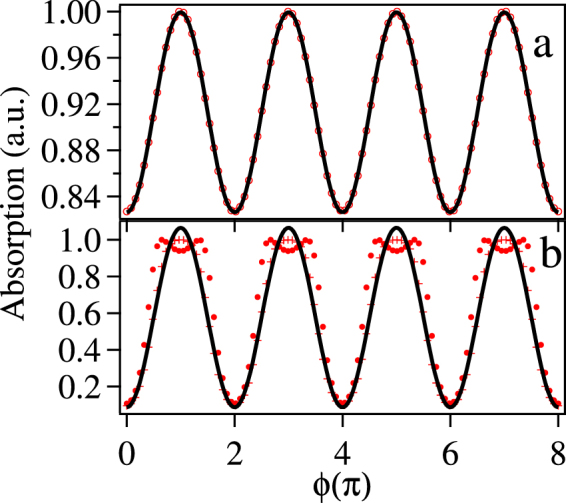
Absorption of the probe laser after thermal averaging in arbitrary scale obtained as (Im(${\rho }_{12}^{{\rm{Thermal}}}$))/max(Im(${\rho }_{12}^{{\rm{Thermal}}}$)) vs phase *ϕ* with *δ*_12_ = *δ*_23_ = *δ*_34_ = *δ*_45_ = *δ*_56_ = *δ*_36_ = 0 and (**a**) $|{{\rm{\Omega }}}_{36}^{{\rm{unk}}}|=0.1{{\rm{\Gamma }}}_{2}$, |Ω_23_| = 2Γ_2_, $|{{\rm{\Omega }}}_{34}^{{\rm{ref}}}|=1.5{{\rm{\Gamma }}}_{2}$, and $|{{\rm{\Omega }}}_{45}^{{\rm{ref}}}|=|{{\rm{\Omega }}}_{56}^{{\rm{ref}}}|=4{{\rm{\Gamma }}}_{2}$. (**b**) crossed points $|{{\rm{\Omega }}}_{36}^{{\rm{unk}}}|=2.5{{\rm{\Gamma }}}_{2}$, |Ω_23_| = 3Γ_2_, $|{{\rm{\Omega }}}_{34}^{{\rm{ref}}}|=2{{\rm{\Gamma }}}_{2}$, and $|{{\rm{\Omega }}}_{45}^{{\rm{ref}}}|=|{{\rm{\Omega }}}_{56}^{{\rm{ref}}}|=4{{\rm{\Gamma }}}_{2}$, solid circled points $|{{\rm{\Omega }}}_{36}^{{\rm{unk}}}|=2.5{{\rm{\Gamma }}}_{2}$, |Ω_23_| = 3Γ_2_, $|{{\rm{\Omega }}}_{34}^{{\rm{ref}}}|=3{{\rm{\Gamma }}}_{2}$, and $|{{\rm{\Omega }}}_{45}^{{\rm{ref}}}|=|{{\rm{\Omega }}}_{56}^{{\rm{ref}}}|=4{{\rm{\Gamma }}}_{2}$.

The probe absorption at room temperature vs the phase *ϕ* with all the detunings to be zero is shown in Fig. 6. From the plot shown with red open circle in Fig. 6a we observe more than 15% change in the probe absorption for the change of the phase from 0 to *π* for the chosen combinations of the control Rabi frequencies. In particular, we have chosen low value of $|{{\rm{\Omega }}}_{36}^{{\rm{unk}}}|=0.1{{\rm{\Gamma }}}_{2}$ and the optimized control fields Rabi frequencies i.e. |Ω_23_| = 2Γ_2_, $|{{\rm{\Omega }}}_{34}^{{\rm{ref}}}|=1.5{{\rm{\Gamma }}}_{2}$, and $|{{\rm{\Omega }}}_{45}^{{\rm{ref}}}|=|{{\rm{\Omega }}}_{56}^{{\rm{ref}}}|=4{{\rm{\Gamma }}}_{2}$. The numerical data points (red open circle) are fitted by a function A + Bsin(f *ϕ* + *θ*), where A, B, f and *θ* are kept as free parameters that yields f = 1 and the fitting is shown with black curve in Fig. 6a. Now, choosing a high value of $|{{\rm{\Omega }}}_{36}^{{\rm{unk}}}|=2.5{{\rm{\Gamma }}}_{2}$ and keeping the other parameters unchanged, we observe more than 80% change in the probe absorption for the change of the phase from 0 to *π* as shown crossed red points, but there is a deviation from sinusoidal behavior. This deviation is compared with the fitted black curve as shown in Fig. 6b. On increasing the value of $|{{\rm{\Omega }}}_{34}^{{\rm{ref}}}|$ to 3Γ_2_ and keeping the other parameters unchanged, there is a splitting of the absorption at *ϕ* = *π* as shown by the solid circled points in this figure.

#### Optimization of sensitivity

Now, we maximize the phase sensitivity for this system for given value of $|{{\rm{\Omega }}}_{36}^{{\rm{unk}}}|$ by using the parameters, Ω_23_, $|{{\rm{\Omega }}}_{34}^{{\rm{ref}}}|$, $|{{\rm{\Omega }}}_{45}^{{\rm{ref}}}|$, and $|{{\rm{\Omega }}}_{56}^{{\rm{ref}}}|$. In order to do this we define a quantity called sensitivity as $S={\rm{Im}}[{\rho }_{12}^{{\rm{Thermal}}}(\varphi =\mathrm{0)}-{\rho }_{12}^{{\rm{Thermal}}}(\varphi =\pi )]/{\rm{Im}}[{\rho }_{12}^{{\rm{Thermal}}}(\varphi =\mathrm{0)}+{\rho }_{12}^{{\rm{Thermal}}}(\varphi =\pi )]$, which is a measure of the phase/strength sensitivity of the system and is to be maximized. For given value of $|{{\rm{\Omega }}}_{36}^{{\rm{unk}}}|$, we maximize the *S* by minimizing 1/*S* or -*S* using matlab inbuilt function “fmincon” treating Ω_23_, $|{{\rm{\Omega }}}_{34}^{{\rm{ref}}}|$, $|{{\rm{\Omega }}}_{45}^{{\rm{ref}}}|$, and $|{{\rm{\Omega }}}_{56}^{{\rm{ref}}}|$ as free parameters but bounded in the region from 0 to 5 Γ_2_. Please note that the values 5Γ_2_ for Ω_23_ $|{{\rm{\Omega }}}_{34}^{{\rm{ref}}}|$, $|{{\rm{\Omega }}}_{45}^{{\rm{ref}}}|$, and $|{{\rm{\Omega }}}_{56}^{{\rm{ref}}}|$ is well in the experimental reach.

**Figure 7:**
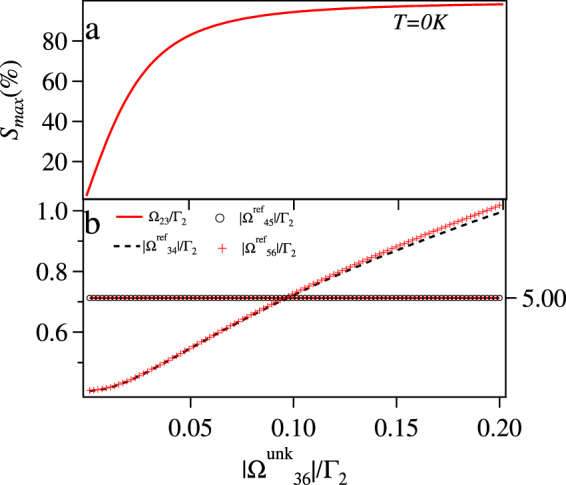
(**a**) The maximum sensitivity *S*_*max*_ (%) vs $|{{\rm{\Omega }}}_{36}^{{\rm{unk}}}|$/Γ_2_ (**b**) The optimum value of $|{{\rm{\Omega }}}_{34}^{{\rm{ref}}}|$/Γ_2_ and $|{{\rm{\Omega }}}_{56}^{{\rm{ref}}}|$/Γ_2_ for *S*_*max*_ (shown by left scale), Ω_23_/Γ_2_ and $|{{\rm{\Omega }}}_{45}^{{\rm{ref}}}|$/Γ_2_ (shown by right scale) vs $|{{\rm{\Omega }}}_{36}^{{\rm{unk}}}|$ for *δ*_12_ = *δ*_23_ = *δ*_34_ = *δ*_45_ = *δ*_56_ = *δ*_36_ = 0 and *T* = 0.

**Figure 8:**
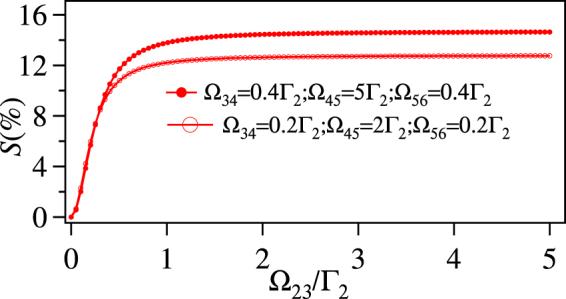
*S*_*max*_ (%) = Im[*ρ*_12_(*ϕ* = 0) − *ρ*_12_(*ϕ* = *π*)]/Im[*ρ*_12_(*ϕ* = 0) + *ρ*_12_(*ϕ* = *π*)] × 100 vs Ω_23_/Γ_2_ for *δ*_12_ = *δ*_23_ = *δ*_34_ = *δ*_45_ = *δ*_56_ = *δ*_36_ = 0, $|{{\rm{\Omega }}}_{36}^{{\rm{unk}}}|=0.005{{\rm{\Gamma }}}_{2}$ and *T* = 0.

We first consider the case without thermal averaging i.e. *T* = 0. The maximized sensitivity, *S*_*max*_ vs $|{{\rm{\Omega }}}_{36}^{{\rm{unk}}}|$ is plotted in Fig. 7(a). The *S*_*max*_ increases with $|{{\rm{\Omega }}}_{36}^{{\rm{unk}}}|$ and starts saturating around 0.05Γ_2_. The corresponding maximizing values of Ω_23_, $|{{\rm{\Omega }}}_{34}^{{\rm{ref}}}|$, $|{{\rm{\Omega }}}_{45}^{{\rm{ref}}}|$, and $|{{\rm{\Omega }}}_{56}^{{\rm{ref}}}|$ are also plotted in Fig. 7(b). The optimum value of the Ω_23_ is as high as possible which is 5Γ_2_ in this case as it is bounded by this limit. This is more clear from the Fig. 8, where *S*_*max*_ increases with Ω_23_ and then saturates around Γ_2_ for any given values of $|{{\rm{\Omega }}}_{34}^{{\rm{ref}}}|$, $|{{\rm{\Omega }}}_{45}^{{\rm{ref}}}|$, $|{{\rm{\Omega }}}_{56}^{{\rm{ref}}}|$, and $|{{\rm{\Omega }}}_{36}^{{\rm{unk}}}|$.

**Figure 9:**
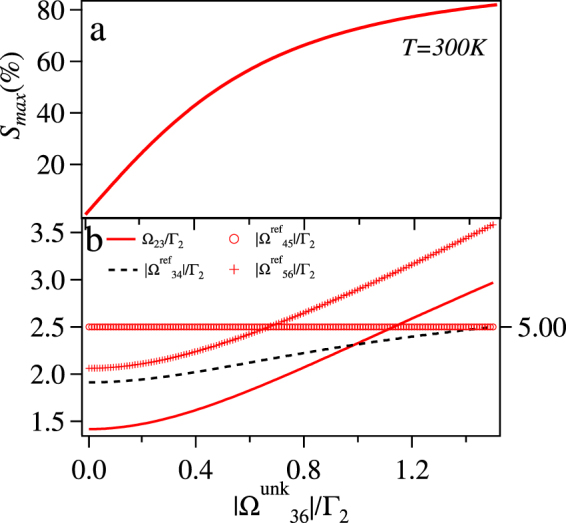
(**a**) *S*_*max*_ (%) vs $|{{\rm{\Omega }}}_{36}^{{\rm{unk}}}|$/Γ_2_ (**b**) The optimum value of Ω_23_/Γ_2_, $|{{\rm{\Omega }}}_{34}^{{\rm{ref}}}|$/Γ_2_, and $|{{\rm{\Omega }}}_{56}^{{\rm{ref}}}|$/Γ_2_ shown by left scale and $|{{\rm{\Omega }}}_{45}^{{\rm{ref}}}|$/Γ_2_ shown by right scale vs $|{{\rm{\Omega }}}_{36}^{{\rm{unk}}}|$/Γ_2_ for *δ*_12_ = *δ*_23_ = *δ*_34_ = *δ*_45_ = *δ*_56_ = *δ*_36_ = 0 and *T* = 300 *K*.

Next, we consider the room temperature case (*T* = 300 *K*), which makes the problem a bit more complicated, as the lineshape of the absorption gets modified significantly as described previously. The maximum sensitivity (*S*_*max*_) vs $|{{\rm{\Omega }}}_{36}^{{\rm{unk}}}|$ is plotted in the Fig. 9(a). The *S*_*max*_ at *T* = 300 *K* is much lower than the case at *T* = 0 as the saturation point is around $|{{\rm{\Omega }}}_{36}^{{\rm{unk}}}|$ = 1.5 Γ_2_ as compared to 0.05Γ_2_ and hence at *T* = 0 the system can detect the phase of lower values of $|{{\rm{\Omega }}}_{36}^{{\rm{unk}}}|$. Unlike the case of *T* = 0, in this case for *S*_*max*_ the value of Ω_23_ ≠ 5Γ_2_ but has optimum values as shown in Fig. 9(b).

### Strength sensitivity

The quantity, *S* defined above can also be used as a measure of the strength/amplitude sensitivity for $|{{\rm{\Omega }}}_{36}^{{\rm{unk}}}|$ for the six-level loopy ladder system. Now we compare the strength sensitivity of the six-level loopy ladder system with the previously studied four-level system^6–11^. The solution of *ρ*_12_ for the four-level system can be obtained from the six-level loopy ladder system by setting $|{{\rm{\Omega }}}_{34}^{{\rm{ref}}}|=|{{\rm{\Omega }}}_{45}^{{\rm{ref}}}|=|{{\rm{\Omega }}}_{56}^{{\rm{ref}}}|=0$ in Eq. 8 and is given by Eq. 11.

11 $${\rho }_{\mathrm{12(4}l)}=\frac{\frac{i}{2}\frac{{{\rm{\Omega }}}_{12}}{{\gamma }_{12}}}{1+\frac{\frac{1}{4}\frac{|{{\rm{\Omega }}}_{23}{|}^{2}}{{\gamma }_{12}{\gamma }_{13}}}{1+\frac{1}{4}\frac{|{{\rm{\Omega }}}_{36}^{{\rm{unk}}}{|}^{2}}{{\gamma }_{13}{\gamma }_{16}}}}$$

The subscript (4*l*) indicates for four-level system. Further the thermal averaging can be done in a similar fashion as in the case of the six-level system i.e. ${\rho }_{\mathrm{12(4}l)}^{{\rm{Thermal}}}=\sqrt{\frac{m}{2\pi {k}_{B}T}}\int {\rho }_{\mathrm{12(4}l)}(v){e}^{-\frac{m{v}^{2}}{2{k}_{B}T}}dv$. We define the strength sensitivity for the four-level system for unknown $|{{\rm{\Omega }}}_{36}^{{\rm{unk}}}|$ as change in the absorption in the presence and the absence of the $|{{\rm{\Omega }}}_{36}^{{\rm{unk}}}|$ normalized by the sum of the two conditions which is mathematically expressed as S = $[{\rho }_{\mathrm{12(4}l)}^{{\rm{Thermal}}}{(|{\rm{\Omega }}}_{36}^{{\rm{unk}}}|\ne 0)-{\rho }_{\mathrm{12(4}l)}^{{\rm{Thermal}}}{(|{\rm{\Omega }}}_{36}^{{\rm{unk}}}|=0)]$/$[{\rho }_{\mathrm{12(4}l)}^{{\rm{Thermal}}}(({|{\rm{\Omega }}}_{36}^{{\rm{unk}}}|\ne 0))+{\rho }_{\mathrm{12(4}l)}^{{\rm{Thermal}}}(|{{\rm{\Omega }}}_{36}^{{\rm{unk}}}|=0)]$. We maximize the sensitivity of the four-level system adapting similar method as for the six-level system but with only one optimizing parameter i.e. Ω_23_.

**Figure 10:**
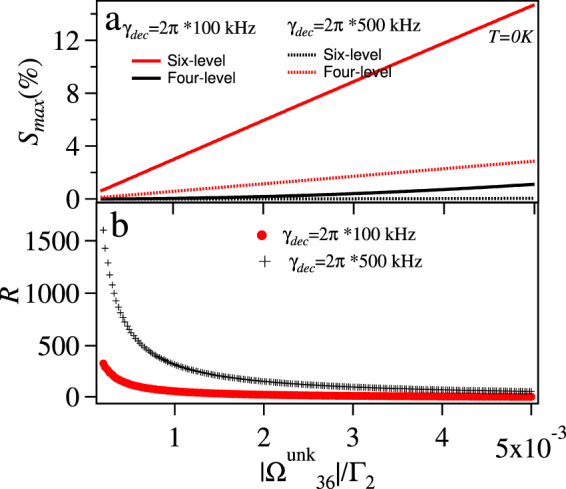
(**a**) *S*_*max*_(%) vs $|{{\rm{\Omega }}}_{36}^{{\rm{unk}}}|/{{\rm{\Gamma }}}_{2}$ for six-level loopy and four-level ladder system (**b**) ratio (*R*) of the sensitivity between six-level and four-level system vs $|{{\rm{\Omega }}}_{36}^{{\rm{unk}}}|/{{\rm{\Gamma }}}_{2}$ at *T* = 0 with all the detunings to be zero and for *γ*_*dec*_ = 2*π* × 100 kHz and *γ*_*dec*_ = 2*π* × 500 kHz.

First, we consider *T* = 0 case. The maximized strength sensitivity for the six-level loopy ladder system and the four-level system is compared in Fig. 10. From this figure it is clear that the six-level system has more sensitivity as compared to the four-level system as shown in Fig. 10(a). In order to quantify this comparison, we plot the ratio of the sensitivities of the six-level to four-level system in Fig. 10(b). The ratio is more for the low values of the $|{{\rm{\Omega }}}_{36}^{{\rm{unk}}}|$. The increased sensitivity for the six-level loopy system is due to the interferometric nature of the system where the effect of small $|{{\rm{\Omega }}}_{36}^{{\rm{unk}}}|$ is enhanced by the large values of the $|{{\rm{\Omega }}}_{34}^{{\rm{ref}}}|$, $|{{\rm{\Omega }}}_{45}^{{\rm{ref}}}|$ and $|{{\rm{\Omega }}}_{56}^{{\rm{ref}}}|$ as the **int** term in Eq. 8 involves multiplication of these quantities. The strength sensitivity of both the systems decreases with increased *γ*_*dec*_ (from 2*π* × 100 kHz to 2*π* × 500 kHz) but the effect is more for the four-level system in comparison to the six-level system as shown Fig. 10b.

**Figure 11:**
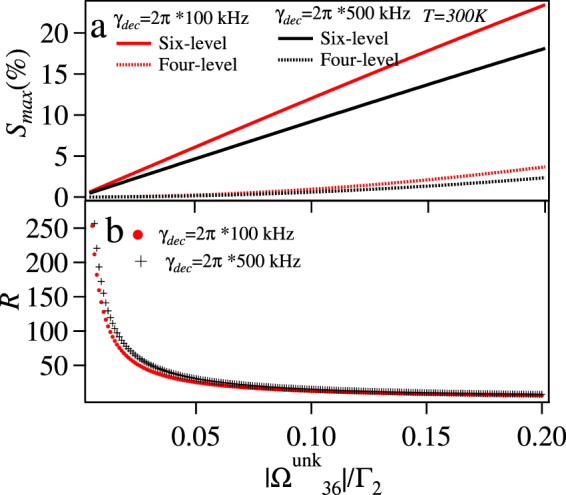
(**a**) *S*_*max*_(%) vs $|{{\rm{\Omega }}}_{36}^{{\rm{unk}}}|/{{\rm{\Gamma }}}_{2}$ for six-level loopy and four-level ladder system (**b**) ratio (*R*) of the sensitivity between six-level and four-level system vs $|{{\rm{\Omega }}}_{36}^{{\rm{unk}}}|/{{\rm{\Gamma }}}_{2}$ at *T* = 300 *K* with all the detunings to be zero and for *γ*_*dec*_ = 2*π* × 100 kHz and *γ*_*dec*_ = 2*π* × 500 kHz.

Now, we consider the case at the room temperature. The strength sensitivity for the six-level and previously studied four-level is plotted in Fig. 11(a). Form this plot it is clear that the six-level system has much superior strength sensitivity as compared to the four-level system. Further we quantify the comparison by plotting the ratio (*R*) of the sensitivities of the six-level to the four-level for different values of $|{{\rm{\Omega }}}_{36}^{{\rm{unk}}}|$ in Fig. 11(b). In order to check the stringency of *γ*_*dec*_ on the sensitivity, we also plot *S*_*max*_ for these two systems taking *γ*_*dec*_ = 2*π* × 500 kHz.

**Figure 12:**
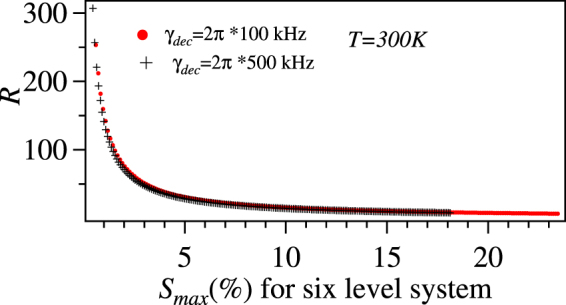
Ratio (*R*) of the sensitivity between six-level and four-level system vs *S*_*max*_ (%) of the six-level system at *T* = 300 *K*. The variation of *S*_*max*_ (%) corresponds to range of $|{{\rm{\Omega }}}_{36}^{{\rm{unk}}}|$ from 0.005Γ_2_ to 0.02Γ_2_.

We also plot the *R* vs maximum sensitivity (*S*_*max*_) of the six-level system which gives the information about the possibility of the detection of $|{{\rm{\Omega }}}_{36}^{{\rm{unk}}}|$. This is an important plot because there is a possibility that the *R* might be huge but can not be detected by the six-level system as well. The detection of *S*_*max*_ up to 1% is very much feasible using locking detection. At this value of sensitivity for the six-level system, the sensitivity of the four-level system will be around $\frac{1}{150}$% as shown in Fig. 12.

Finally one more important point is that, for the six-level loopy ladder system the MW field $|{{\rm{\Omega }}}_{36}^{{\rm{unk}}}|$ can be detected by just varying the phase of the reference MW fields, while in the case of the four-level system we need to insert and remove MW mechanical shield.

### Frequency range

The frequency range of the atomic based MW interferometry can be any where from the range of the few tens of MHz, GHz and THz. The rydberg states can be chosen depending upon the interest of the frequency region of MW field. For example, for frequency in the range of few tens of GHz n _*ryd*_’s should around 54^7^ while for tens of MHz it should be higher number and it is around 57 in case of Cs^9^. For THz regime this should be around 20^33^.

## Discussion

In conclusion we theoretically study a six-level loopy ladder system using Rydberg states for the phase sensitive MW or RF electrometry. This is based upon the interference between the two sub-systems of EITATA. In counter-propagating configuration of the probe and control laser there is a change of the lineshape of the probe absorption due to Doppler averaging. The limitation of the proposed system is the decoherence rate between the ground state and the Rydberg states but not the thermal Nyquist noise as in the case of the electrical circuit based MW interferometry. The previously explored four- level atomic system has the same limitation and is already much superior than the electrical circuit for the strength sensitivity, frequency range and spatial resolution. This proposed system further improves the sensitivity by two orders of magnitude, removes the drawback of the phase insensitivity of the previous atomic four level-system and retains the advantages of the large frequency range of operation and spatial resolution. This system provides a great possibility to characterize the MW or RF electric fields completely including the propagation direction and the wavefront. This work will be quite useful for MW and RF engineering hence in the communications specially in active radar technologies and synthetic aperture radar interferometry.
